# Long-term, high-dose opioid prescription for chronic non-cancer pain in primary care: an observational study

**DOI:** 10.3399/BJGPO.2021.0217

**Published:** 2022-09-07

**Authors:** John Bailey, Simon Gill, Rob Poole

**Affiliations:** 1 Centre for Mental Health and Society, Bangor University Wrexham Academic Unit, Technology Park Wrexham, Wrexham, UK; 2 Betsi Cadwaladr University Health Board, North Wales, UK; 3 Centre for Mental Health and Society, Bangor University, Bangor, UK

**Keywords:** Opioid analgesics, Chronic pain, Drug prescriptions, Primary care, Oxycodone, Morphine

## Abstract

**Background:**

Opioid prescriptions for chronic pain have risen sharply over the last 25 years; harms associated with these drugs are related to dose and length of use.

**Aim:**

The main aim of this study was to identify patients prescribed long-term, high-dose (LTHD) opioids in the community and to assess the prevalence of such use.

**Design & setting:**

An observational study of opioid prescribing in two demographically dissimilar GP practices in North Wales, UK.

**Method:**

Details of opioid prescriptions were collected for 22 841 patients, of whom 1488 (6.5%) were being prescribed opioids on the census date. Exhaustive examination of the data identified all patients who were prescribed oral morphine equivalent doses of ≥120 mg/day for ≥1 year.

**Results:**

All these patients were being prescribed ≥120 mg/day, as a single drug, morphine, oxycodone, fentanyl, or buprenorphine, irrespective of opioid polypharmacy. Across both practices, 1.71/1000 patients were identified as LTHD users of opioid medication for chronic non-cancer pain (CNCP). Prevalence was similar in the two practices. Repetition of the process until January 2021 showed no change in the pattern.

**Conclusion:**

This study offers confirmation that a significant group of patients are prescribed long-term opioid medication for chronic pain at doses that are unlikely to be effective in reducing pain, but are likely to have harmful consequences. The findings offer a simple, reliable, and practical method of data extraction to identify these patients individually from routinely collected prescribing data, which will help in monitoring and treating individuals and establishing the problem prevalence.

## How this fits in

While there is much concern about the increasing prescribing of opioid medication in recent years, little is known about those patients prescribed high doses in the long term. A detailed examination of prescribing for CNCP at the individual patient level informed the development of a simple method for identifying those prescribed sustained high doses. This allows for the identification for monitoring and intervention of those patients most likely to be at risk of harm resulting from opioid doses that are likely to be ineffective in reducing pain.

## Introduction

UK opioid prescribing has increased substantially since 1990,^1,2^ with similar changes internationally,^3^ especially in North America.^4–6^ There is widespread concern in the UK related to severe problems in the USA,^7–10^ where there has been an epidemic of opioid use and resulting harm arising, in part, from the indiscriminate prescribing and diversion of pain medication. There is little evidence for problems of the scale and severity experienced in the USA occurring outside North America. Most opioid prescribing in the UK is for CNCP^11–13^ despite a lack of evidence of effectiveness^14–19^ and the risk of direct and indirect harms.^15,17,19–26^ Epidemiological evidence shows that opioids for CNCP are associated with worse functioning and quality of life,^27–30^ and the authors' clinical experience suggests this is particularly true with high doses.

An emerging consensus suggests that opioid treatment for chronic pain should be approached with care rather than with unbridled enthusiasm.^31^ However, the field must now cope with the consequences of past over-enthusiasm. It is important to find practical ways to identify patients in primary care on LTHD regimens.

Studies of opioid prescribing in the UK are summarised in Table 1. Overall prescribing may have decreased but rates remain much higher than they were 25 years ago.^2,11,32^ In contrast to the decrease in overall prescribing, the prescribing of stronger drugs has increased,^2,11,33,34^ as has the number of people prescribed long-term opioids.^32,35^ These studies illustrate persistent concerns about use of opioid medication in the UK population. However, to properly understand and then address the problem, individual-level information is needed. Aggregated population-level statistics showing 100 prescriptions issued cannot distinguish between 100 people with one prescription each, 10 people with 10 prescriptions each, or one person with 100 prescriptions, and each of these indicates a different clinical situation. There is also a need for a method to identify individual patients on these regimens within practice populations to facilitate evaluation and intervention.

**Table 1.** UK studies of opioid prescribing in primary care

Study	Study period	Location	Study population	Findings
NTA (2011)^1^	1991–2009	England	All prescriptions	Five-fold increase in prescribing opioids.Regional variations in prescribing.
Ruscitto *et al* (2015)^46^	1995–2010	Tayside	Primary care	Increase in opioid prescribing.Larger increase in “strong”^a^ opioid prescribing.Associated factors: polypharmacy, social deprivation.
Curtis *et al* (2019)^2^	1998–2018	England	Primary care	Increases in prescribing between 1998 and 2016.Increase in ME amount prescribed much greater.Prescriptions decreased after 2016.Associated factor: geographical variation.
Zin *et al* (2014)^13^	2000–2010	England	Primary care	“Huge” increase in “strong”^a^ opioid prescribing.Majority (88%) for non-cancer pain.
Cartagena Farias *et al* (2017)^35^	2000–2015	England	Primary careNon-cancer pain	Increasing numbers of people prescribed opioids.Prescribing for longer periods.Associated factors: age, social deprivation, regional variation.
Bedson *et al* (2016)^48^	2002–2013	England	Primary careMusculoskeletal pain	Long-term prescribing increased to 2009; slight decrease after 2011.Increased prescribing of long-acting opioids.
Green *et al* (2012)^49^	2004-2007(?)^b^	North Staffordshire	Primary careJoint painAged >50	Factors associated with increased rates of prescription.Factors associated with “strong”^a^ opioid use.
Foy *et al* (2016)^33^	2005–2012	West Yorkshire	Primary careNon-cancer pain	Prescribing of weaker opioids doubled.Six-fold increase in “stronger”^a^ opioid prescribing.Patient and prescriber factors associated with stepping up to “stronger” opioids.
Davies *et al* (2019)^11^	2005–2015	Wales	Primary careNon-cancer pain	Large increase in prescribing of “strong”^a^ opioids.Associated factors: age, social deprivation, anxiety or depression diagnosis.
Jani *et al* (2020)^50^	2006–2017	England	Primary care	Increased prescribing of opioids: codeine, morphine, tramadol, oxycodone.Initiated high doses tend to be maintained.Associated factors: social deprivation, regional variation, polypharmacy.
Mordecai *et al* (2018)^34^	2010–2014	England	Primary care	Increase in amount (in ME) prescribed.Associated factors: social deprivation; regional variation.
Ponton & Sawyer (2018)^36^	2012(?)^b^	South East England	Primary carePatients prescribed high doses	Identified patients prescribed doses ≥120 mg ME of “strong”^a^ opioids as candidates for specialist input.
Ashaye *et al* (2018)^51^	2011–2012	London and Midlands	Primary careMusculoskeletal pain	Long-term prescribing common.Possible overprescribing in more than a quarter of patients receiving “strong”^a^ opioids.
Public Health England (2019)^32^	2015–2018	England	Primary care	Prescriptions and proportion of population prescribed opioids declining from historically high rates.High rates of long-term prescribing.Associated factor: social deprivation, polypharmacy.
Bastable & Rann (2019)^37^	2018	East England	Primary carePatients prescribed high doses	Identified patients prescribed opioid doses ≥120 mg ME.Co-prescribing of Z-drugs, benzodiazepines, and gabapentinoids.

ME = Morphine equivalent..

^a^ Drugs categorised as “strong” vary between studies, but always include morphine, oxycodone, and fentanyl.

^b^ This is estimated from the published text, which does not indicate the data collection period.

The aim of this study was to develop a practical method to identify all patients on LTHD opioid regimens within primary care and to describe patterns of opioid prescribing in two practice populations.

## Method

The criteria for LTHD opioid prescribing were 1) daily oral morphine equivalent (ME) dose of 120 mg or more;^36,37^ 2) prescribed continuously for more than 1 year; and 3) patients with cancer were excluded. The Royal College of Anaesthetists^38^ indicates that *‘the risk of harm increases substantially at doses above an oral ME of 120mg/day, but there is no increased benefit‘* and the British Pain Society^39^ has recommended that patients with CNCP being prescribed opioids at this doses or above should be referred to specialist pain services. A one-year minimum prescribing duration was chosen as it is unequivocally long-term use.

A point prevalence study was carried out in two GP practices in North Wales. Practice A was a large, fully medically staffed practice in a market town with national average levels of social deprivation, as indicated by the Welsh Index of Multiple Deprivation.^40^ It was not identified, either internally or externally, as an outlier regarding opioid prescribing, lying below the mean for the Local Health Board (LHB).

During data collection in practice A, the authors were asked to provide support on opioid prescribing to a second GP practice, practice B, in an ex-mining community with higher Welsh Index of Multiple Deprivation^40^ levels of social deprivation. It was entirely staffed by locum GPs and administered by the LHB. Opioid prescribing was close to the LHB mean.

Data were extracted from the practices’ computerised records for all prescriptions of opioid analgesic medication, including compound drugs (see Box 1 for details), dated between 1 October 2016 and 31 January 2017 (practice A), and 17 January and 15 May 2017 (practice B). The data included: age and sex; date of prescription; drug name, strength, and dose direction; and quantity prescribed. The weeks beginning Monday 9 January 2017 (practice A), and Monday 24 April 2017 (practice B), were selected as the index week and all prescriptions issued in that week or for use in that week were identified. PRN (as required) prescriptions dated in the month preceding the index week and/or records indicated continuing use, were included. An estimate of daily dose was made. For PRN prescriptions, this was calculated by dividing the total quantity of drug prescribed by the number of days between the two prescriptions nearest the index week. This method was also used where there was a clear difference between the use calculated and the amount specified in the dose direction; otherwise the dose as directed was used. Oral ME doses were calculated using conversion tables from Palliative Care Guidelines Plus;^41^ total 24-hour doses were calculated for individual drugs and for all opioids issued.

Box 1 **Searching practice records for prescriptions for opioid medication .**

The two GP practices studied in this research used different database systems (Vision and EMIS) to record practice information. Different search terms were enabled in these systems to access information about drug prescriptions.

In the Vision system, the search terms were:

Drug Class of type *Opioid Analgesics*, and
Drug Class of type *Non-opioid and Compound Analgesics*.

In the EMIS system, the search terms were:

The Drug is *Opioid Analgesics*, and
The Drug is *Compound Analgesic Preparations*.

These searches produced slightly differing results, but included, in both cases, all prescriptions for drugs listed by BNF [British National Formulary] as opioid analgesics, in the relevant time period. Therefore, there were false positive results but no false negatives.

It was assumed that there were likely to be three ways in which total ME dose might exceed 120 mg/day:

- as a high dose of a single drug;
- as a relatively high dose of one drug with a dose of a second drug;
- as lower doses of several opioid drugs in combination.

Three sets of criteria were applied sequentially to the collected data to identify three (non-mutually exclusive) patient groups:

- those prescribed a daily dose of a single drug ≥120 mg ME;
- those prescribed a daily dose of a **single drug** between 60 mg ME and 119 mg ME and other opioid(s);
- those prescribed three or more different opioids. (This iteration identified very few cases not found in A or B, none of which had doses approaching 120 mg ME. It was subsequently discontinued.)

All patients prescribed LTHD opioids were identified. Those with a cancer diagnosis were excluded. Comparison data on these patients for the corresponding period a year earlier were collected.

In practice A, additional data were collected from review of patient records for those prescribed LTHD opioids: indication for use of opioid; non-opioid analgesic or adjuvant medications; referral history to secondary care pain services; and longitudinal opioid prescription histories.

The LHB policy is that opioid replacement for substance misuse must be prescribed by specialist services, not GPs; no opioid prescriptions for substance misuse were identified.

When the data had been collated, the findings were presented to a meeting of practice A's staff. Prescribers’ comments were elicited and recorded. This was not possible in practice B due to discontinuity of medical staffing.

The exercise was repeated yearly in practice A in the corresponding week from 2018–2021. One repeat audit was conducted in practice B in October 2020.

Service users from PÂR-NCMH^42^ were consulted during the planning of this research.

## Results

Table 2 summarises opioid prescribing data from the two primary care practices. In both practices, all patients identified as having been prescribed LTHD opioids reached the threshold dose through prescription of a single drug at ≥120 mg ME. No patient crossed the dose threshold only when doses of different opioids were aggregated.

**Table 2.** Opioid prescribing in Practice A and Practice B in the index week 2017

	Practice A	Practice B	Both practices
Number of patients in practice (all ages)	14 355	8486	22 841
Prescriptions for opioids: including compound drugs	914	734	1648
non-compound drugs	389	275	664
Patients prescribed opioids: including compound drugs	821 (5.7%)^a^	667 (7.9%)^a^	1488 (6.5%)^a^
non-compound drugs	337 (2.3%)	246 (2.9%)	583 (2.6%)
Patients with estimated daily dose ≥120 mg ME	34^b^ (2.37)^c^	19 (2.24)^c^	53 (2.32)^c^
as above + use for more than 1 year	**25** (**1.74**)	**14** (**1.65**)	**39** (**1.71**)
Patients with dose ≥60 mg & <120 mg ME in one drug	28^d^ (1.95)	21 (2.47)	49 (2.15)
as above + other opioid(s)	15 (1.04)	4 (0.47)	19 (0.83)

^a^% of all practice patients prescribed these drugs. ^b^In this practice there were an additional 4 patients with a cancer diagnosis. ^c^Number per thousand patients. ^d^In this practice there was 1 additional patient with a cancer diagnosis.

Results in bold are the main findings of the study.

Combining figures from the two practices showed a LTHD opioid prevalence of 1.71 per 1000 patients (practice A: 1.74 per 1000; practice B: 1.65 per 1000)

All the patients on LTHD opioids were prescribed morphine, oxycodone, or fentanyl transdermal patches. In **practice A**:

- eight on morphine (highest daily ME 619 mg),
- six on oxycodone (highest daily ME 480 mg),
- eleven on fentanyl (highest daily ME 540 mg).

Fourteen out of 25 also had PRN opioid prescriptions. When PRN was added, the highest daily ME was 778 mg.

In **practice B**:

- four on morphine (highest daily ME 480 mg),
- five on oxycodone (highest daily ME 320 mg),
- five on fentanyl (highest daily ME 540 mg),

Three out of 14 patients also had PRN opioid prescriptions.

Table 3 summarises high dose opioid prescribing in practice A between 2017 and 2021. LTHD opioid rates increased from 1.74 per 1000 patients to 1.92 per 1000 patients during this period. In practice B, this rate decreased from 1.65 per 1000 patients to 1.26 per 1000 patients in 2020. Supplementary Table S1 shows that once high doses were reached they tended to be maintained, and that in January 2021 there were a small number of LTHD buprenorphine prescriptions (*n* = 3; 6% of all the patients prescribed LTHD opioids from Practice A).

**Table 3.** Longitudinal high-dose prescribing data from Practice A

	**2017**	**2018**	**2019**	**2020**	**2021**
Registered patients	14 355	14 584	14 797	15 809	16 140
≥60<120 mgME	27 (1.88)^a^	25 (1.71)^a^	24 (1.62)^a^	23 (1.45)^a^	24 (1.49)^a^
≥120 mgME	34 (2.37)^a^	37 (2.54)^a^	37 (2.50)^a^	36 (2.28)^a^	36 (2.23)^a^
≥120 mgME >1 year	25 (1.74)^a^	28 (1.92)^a^	30 (2.03)^a^	32 (2.02)^a^	31 (1.92)^a^

^a^ *n* per 1000 registered patients.

In **practice A**, the age of patients prescribed LTHD opioids ranged from 32 to 88 years (mean 61.1, median 58); 15 (60%) were female. In **practice B**, ages ranged from 30 to 80 years (mean 51.7, median 44.5); five (36%) were female. Table 4 gives detailed age distributions.

**Table 4.** Age profile of patients prescribed LTHD opioids in the two practices in the index week 2017

	Age, years							
	0–24	25–34	35–44	45–54	55–64	65–74	75–84	≥85
**Practice A**								
Patients in age group, *n*	4006	1729	1735	2404	1772	1524	902	283
% of all patients in age group	28	12	12	17	12	11	6	2
Long-term high dose users, *n*	0	1	2	7	5	3	5	2
*n*/1000 patients	0	0.58	1.15	2.91	2.82	1.97	5.54	7.07
**Practice B**								
Patients in age group, *n*	2632	1170	1119	1236	972	836	410	106
% of all patients in age group	31	14	13	15	11	10	5	1
Long-term high dose users, *n*	0	2	4	3	3	0	3	0
*n*/1000 patients	0	1.71	3.57	2.43	3.09	0	7.32	0
**Combined**								
Patients in age group, *n*	6638	2899	2854	3640	2744	2360	1312	389
% of all patients in age group	29	13	12	16	12	10	6	2
Long-term high dose users, *n*	0	3	6	10	8	3	8	2
*n*/1000 patients	0	1.03	2.10	2.75	2.91	1.27	6.10	5.14

**Practice A** data showed indications for opioids: musculoskeletal pain in *n* = 21/25 and chronic abdominal pain in *n* = 4/25. In Practice A, *n* =9/25 had never been referred, at any point, to secondary care pain services and *n* = 19/25 were prescribed non-opioid analgesics or adjuvant medications:

- amitriptyline *n* = 7,
- other antidepressants *n* = 11 (2 antidepressants in two instances),
- benzodiazepines *n* = 4,
- hypnotics *n* = 1,
- gabapentinoids *n* = 7 (1 patient with a diagnosis of epilepsy),
- non-steroidal anti-inflammatory drugs *n* = 4,
- ketamine *n* = 1.

It was possible to determine the duration of continuous opioid use for *n* = 23/25 patients (the other two patients were on high-dose opioids on registration with the practice). Regular non-compound opioids were initiated 42–240 months before the index week (median 126). Initiation of doses ≥120mg ME was 13–198 months before the index week (median 106).

Findings from the first year’s data were presented to a group of GPs and other staff at Practice A . They expressed surprise at the number of patients prescribed LTHD opioids, having expected there to be few or none. The consensus was that patients experience adverse drug effects at doses below 120 mg ME per day, and that a lower dose-threshold might be more appropriate. Problems with adjuvant medications were raised. Conversations about opioids were described as difficult for prescribers and patients, sometimes leading to conflict. Refusal to increase dose or strength and raising cessation or reduction of opioids were difficult topics. There was a perceived lack of alternatives. Prescribers did not feel they had full control of opioid prescribing. The GPs were asked if there were any patients prescribed LTHD opioids who experienced satisfactory levels of pain relief without negative effects; none could be identified.

## Discussion

### Summary

This study's findings show that LTHD opioid use for CNCP can be identified in primary care records by searching for prescriptions of morphine, fentanyl, oxycodone, or buprenorphine at or above 120 mg ME per day and examining prescriptions 1 year previously. This method (see Box 2) is less laborious than that used in previous UK studies.

Box 2 **Procedure for identifying patients prescribed LTHD opioids from routinely collected prescription data.**

1. Identify all patients prescribed morphine, oxycodone, fentanyl, or buprenorphine.
2. From 1, identify all patients prescribed one of these drugs at ≥120 mg ME daily dose.
3. From 2, identify patients prescribed these drugs and these doses 1 year previously.

As indicated in Table 2, examination of opioid prescribing data for practices A and B identified all patients prescribed LTHD opioids. A rate of 1.71 per 1000 patients was calculated for the practices combined. Unadjusted extrapolation would suggest that there might be over 100 000 patients prescribed LTHD opioid medication in the UK, but this figure is not epidemiologically reliable; it is a crude estimate. These patients are at risk of harm and functional impairments with very limited pain relief.

This study's criteria were conservative: most of the literature accepts 120 mg ME as the threshold for limited analgesic benefit and high risk of harm.^38^ A one-year duration was chosen pragmatically. It is likely that some patients experience negative effects at lower doses and shorter durations. Table 2 sets out data for high doses for less than 1 year and daily doses between 60 and 120 mgME.

This study shows that some patients follow LTHD regimens for many years. No judgements about drug effectiveness can be made from this study's findings, but there is a real possibility that harms outweigh benefits for these patients.

This study found frequent use of adjunctive pain medications alongside high dose opioids. Many of these have sedative effects that compound the effects of opioids.

The feedback meeting with practice A's medical staff suggested that LTHD prescribing can arise without the prescriber intending it, confirming uncertainty over the locus of control. The discussion confirmed previous findings regarding difficult doctor–patient interactions about opioids.^43,44^ The prescribers were unable to identify any patients prescribed LTHD opioids who experienced good pain relief and no adverse effects on functioning.

### Strengths and limitations

This study’s main limitation was sample size. Small-scale studies are susceptible to sampling error, and the findings should not be over-interpreted. This study has presented purely descriptive findings. Set against this, the size of the study allowed collection and analysis of detailed data on an individual patient level with direct counts of cases rather than estimates from a sample of the population of interest. This is a necessary step in developing practical methods to generate large individual level datasets and to identify individual patients for medication review. Replication in other locations with different demographics would be helpful. The authors are particular aware of the lack of ethnic diversity in the area studied; 97.8% White in North Wales in 2015-2017.^45^ These findings are indicative, but a larger study is necessary for fully generalisable findings. The method outlined in this study will facilitate larger scale studies of LTHD opioid use.

### Comparison with existing literature

Two previous studies sought to identify patients prescribed high-dose opioids for CNCP.^36,37^ Their methods did not measure duration, and made extensive demands on clinical and administrative staff time. This study's method was less laborious and captures the group of greatest concern.

Rates of LTHD prescribing in practice A are consistent with other evidence on potent, high-dose and long-term opioid prescribing.^2,11,32,34,35^ Increasing levels of such prescribing in practice A suggest that prescriber awareness of LTHD opioids because of this study was insufficient to alter prescribing. Decreased LTHD prescribing in practice B may be a result of pharmacist prescribing support provided in the absence of permanent medical staff.

The number of patients prescribed opioids, sex ratio, and average age in practice A are similar to a recent primary care study.^35^ The results for practice B are different. Research has shown that higher overall rates of opioid prescribing are associated with older-age patients.^11,13,35^ Table 4 shows higher *rates* in people over 75 years, though the absolute *number* is evenly spread across those aged over 40 years.

Research has shown higher levels of opioid prescribing in deprived areas.^2,11,32,34,35,46^ Accordingly, rates of overall opioid prescribing were higher in the more socially deprived practice B. However, rates of LTHD opioid prescribing were similar in both practices. The prevalence finding in this study was higher than in previous studies. This may reflect differences in method, geographical differences, change in prevalence over time, or all three. However, it does not suggest a recent reduction in high-dose opioid prescribing. It underlines the need for evidence-based interventions to reduce high-dose prescribing.

### Implications for research and practice

This study's method is practical and reliably identifies patients prescribed LTHD opioids in UK primary care. It has utility in auditing such regimens and in identifying at-risk patients. It has potential to unpick the fine grain epidemiology of the problem.

The findings indicate factors that should be explored in future studies: social deprivation, age, duration of high-doses regimens, indications for opioids, adjunctive medications, and better prescribing strategies. Such studies will be important in the development of better pain management strategies in primary care (see^47^).

## Data

The dataset relied on in this article is available from the corresponding author on reasonable request.
